# Urban-rural disparity in body mass index: is dietary knowledge a mechanism? Evidence from the China Health and Nutrition Survey 2004-2015

**DOI:** 10.7189/jogh.13.04064

**Published:** 2023-07-28

**Authors:** Liang Ma, Philip J Schluter

**Affiliations:** 1 The First Hospital of China Medical University, Shenyang, China; 2 Faculty of Health, University of Canterbury, Christchurch, New Zealand; 3 School of Clinical Medicine, Primary Care Clinical Unit, The University of Queensland, Brisbane, Australia

## Abstract

**Background:**

The co-existence of undernutrition and overweight/obesity has been recognised as a severe challenge in China, with substantial urban-rural disparity. We evaluated short- and long-term associations of urban-rural locality on body mass index (BMI) in Chinese adults overall and stratified by sex, focusing on whether dietary knowledge plays a mediating role.

**Methods:**

We used cross-sectional and longitudinal study designs with structural equation modelling based on the 2004 (T1) and 2015 (T2) waves of the China Health and Nutrition Survey. We adjusted the models for covariates and performed sensitivity analyses.

**Results:**

We cross-sectionally analysed 8932 (53.1% women) and 11 216 adults (54.3% women) at T1 and T2, respectively, and longitudinally investigated 4073 adults (55.6% women) in both T1 and T2. The estimated average dietary knowledge and mean BMI increased from T1 to T2. At each time point, we found significant indications of direct (e.g. urban-rural locality to BMI, urban-rural locality to dietary knowledge, and dietary knowledge to BMI) and indirect associations (e.g. urban-rural locality to dietary knowledge to BMI) overall and for men and women (except that urban-rural locality to BMI) separately. The long-term association between urban-rural locality and BMI attenuated over time and was not mediated by dietary knowledge change alone. Nevertheless, dietary knowledge interacted with BMI, which acted as a pathway from urban-rural locality to BMI in the long term.

**Conclusions:**

Urban-rural disparity in BMI persists in Chinese adults and is mediated by dietary knowledge. Policy and educational efforts to improve dietary knowledge among rural people may reduce China’s urban-rural disparity in BMI.

The double burden of malnutrition (DBM), meaning a coexistence of undernutrition and overweight/obesity, affects many low- and middle-income countries (LMICs) [1], including China, where it has an estimated prevalence of 44% among adults [2]. This trend appears to be worsening. In 2018, an estimated 8.1% of Chinese adults were living with obesity compared to 3.1% in 2004 [3], contributing to 11.1% of deaths associated with non-communicable diseases [4]. Simultaneously, over half of adults have dietary intake less than the Chinese estimated average requirement for key micronutrients [2]. Besides worse health status, DBM imposes substantial economic burdens on healthcare systems [5,6]. The annual obesity-related cost in China between 2000 and 2009 was estimated at ¥24 billion (2.5% of national medical cost) [7], and is projected to increase to ¥418 billion (22% of national medical cost) by 2030 [8].

Body mass index (BMI) is a routinely used epidemiological anthropometric marker for body size and DBM classifications. Consistent with a social determinations of health framework [9], urban-rural locality has been recognised as an important determinant of BMI [10-12]. Although extensively studied [13-16], the pathways leading to BMI differences between urban and rural locations remains poorly understood. They are likely mediated by proximal factors such as diets and physical activity [17,18]. However, few studies have examined other distal factors, such as dietary knowledge, as potential mediators in this association. Individuals living in urban areas have demonstrated better dietary knowledge than those in rural areas [19]. According to the knowledge, attitude, and practice model [20], this knowledge likely influences diets, a key proximal factor associated with BMI [21].

Despite the plausible associations between urban-rural locality, dietary knowledge, and BMI, current literature is limited in several ways. The association between dietary knowledge and BMI varies substantially between studies, which in turn usually apply cross-sectional designs, leading to calls for longitudinal data to explore the factors and mechanisms of malnutrition [22-24]. Furthermore, despite the relevance of sex differences [3,10], there are no sex-stratified analyses investigating urban-rural locality, dietary knowledge, and BMI.

To address the current gap in the literature, we evaluated several mediation models that specified the relationship between urban-rural locality, dietary knowledge, and BMI, both overall and stratified by sex, hypothesising that urban-rural locality is associated with BMI, which is in turn mediated by dietary knowledge and may be different between men and women.

## METHODS

### Study design

We used both a cross-sectional and a longitudinal design with data from the 2004 (when dietary knowledge was first included) and 2015 (the latest) waves of the China Health and Nutrition Survey (CHNS), a cohort launched through an international collaboration between the Chinese Center for Disease Control and the Carolina Population Center at the University of North Carolina at Chapel Hill to understand how the wide-ranging social and economic changes in China are affecting a wide array of nutrition and health-related outcomes [25]. The survey has been conducted multiple times between 1989 and 2015. The full sample was drawn from 15 provinces and municipal cities using a multistage cluster sampling scheme (individuals, households, and communities).

### Study population

**Figure 1:**
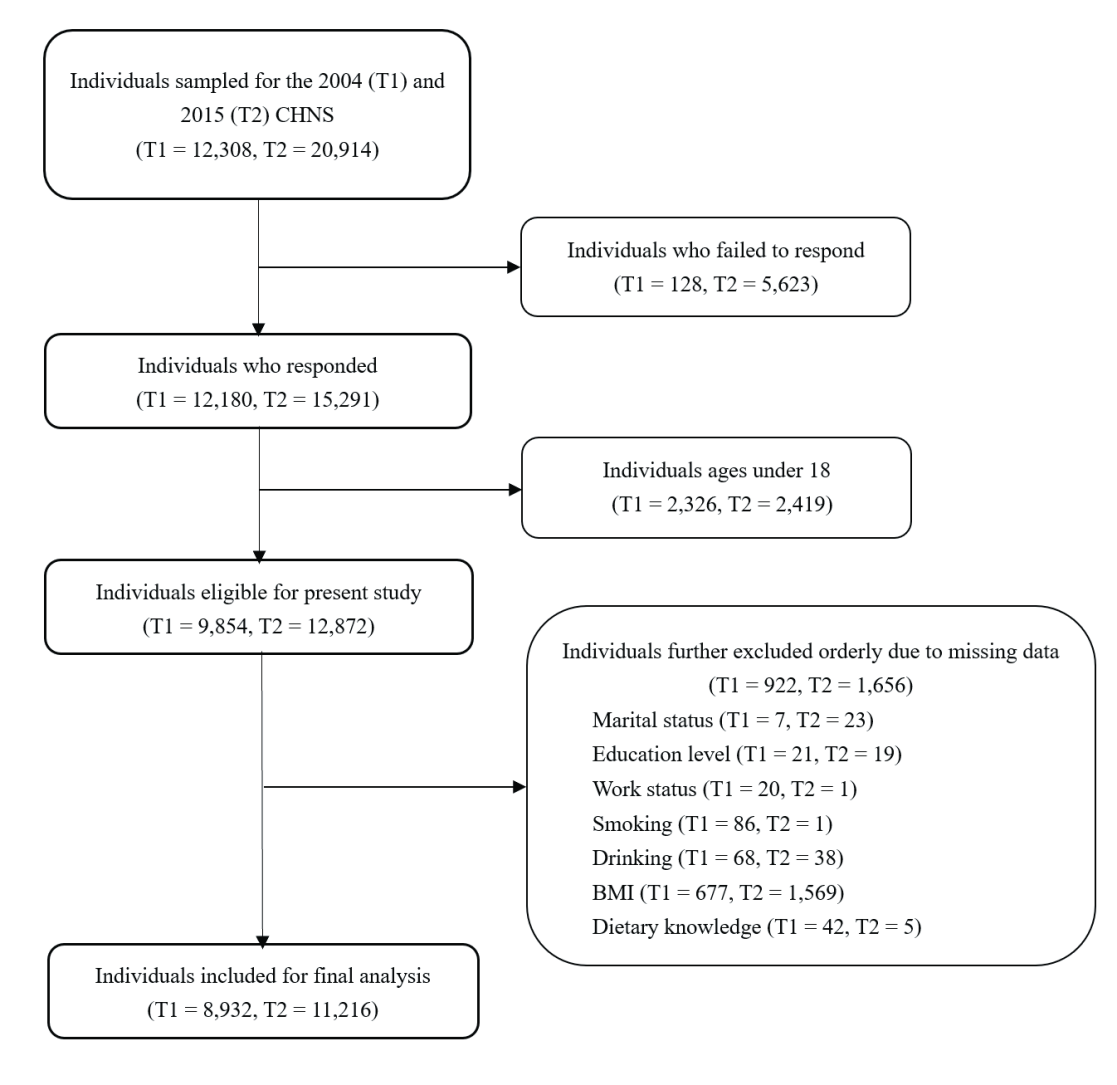
Flowchart of study participants.

There were 12 308 and 20 914 individuals sampled at 2004 (T1) and 2015 (T2), respectively, with 12 180 (98%) and 15 291 (73%) agreeing to participate at each time point. Because children and adolescents may differ significantly from adults in cognitive ability and cause of malnutrition, we excluded individuals aged <18 years (T1: n = 2326; T2: n = 2419). We also excluded participants who had missing data for one or more of the primary variables (T1: n = 922; T2: n = 1656). The final sample included 8932 participants (53.1% women) at T1 and 11 216 (54.3% women) at T2. Of these, 4073 adults (55.6% women) participated at both time points and were included in the longitudinal analyses (**Figure 1**).

### Urban-rural locality, dietary knowledge, and BMI

There are no universal schemes for categorising urban and rural locations to date. The dichotomous definition of urban and rural locations might fail to account for variations within an urban or rural location, such as suburban areas [26]. The CHNS questionnaire employed four strata of different areas (city, town/county capital city, suburban, and rural village). We accordingly categorised participants from cities, towns, or county capitals city neighbourhoods as urban and those from suburban or villages as rural.

The CHNS measured dietary knowledge using 12 diet-related items at T1 (Table S1 in the **Online Supplementary Document**) and 17 at T2. For consistency, we used the same items in 2004 to indicate dietary knowledge in 2015. Each item was rated on a five-point Likert scale ranging from 1 (strongly disagree) to 5 (strongly agree), with 3 being neutral or “unknown”. Based on the World Health Organization (WHO) development of food-based dietary guidelines for the Asian region, we indicated these items as “True” if they met the criteria and “False” if not [27,28], and reverse-coded the latter to ensure consistency with the true items. All the items were summed, and a greater total score reflected a higher level of dietary knowledge.

BMI is defined as the weight in kilograms divided by the square of the height in meters (kg/m^2^). We considered BMI<18.5 kg/m^2^ as undernutrition, 18.5-24.9 kg/m^2^ as normal, 25.0-29.9 kg/m^2^ as overweight, and ≥30 kg/m^2^ as obese [29].

### Covariates

We selected the following covariates from the CHNS questionnaire based on available literature on determinants of BMI and dietary knowledge: age, sex, marital status, educational level, work status, cigarette smoking, and drinking of alcohol. Cigarette smoking was measured through responses to the question “How many cigarettes do you smoke per day?”, with responses categorised into non-smoking, 1-3 cigarettes/d, and ≥3 cigarettes/d. Drinking of alcohol was measured by responses to the question “How often did you drink beer or any alcoholic beverage?”, with responses categorised into non-drinking, light drinking (including “no more than once a month”, “once or twice a month”, and “once or twice a week”), and heavy drinking (including “3-4 times a week”, and “almost every day”). We entered sex, marital status, education level, and work status as dichotomous variables (e.g. men vs women, married vs unmarried, non-college vs college, and employed vs unemployed), but retained age as a continuous variable.

### Statistical analysis

**Figure 2:**
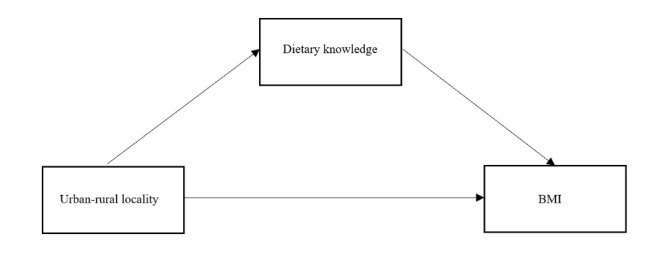
Mediation model 1: dietary knowledge with urban-rural locality and BMI at T1 and T2.

**Figure 3:**
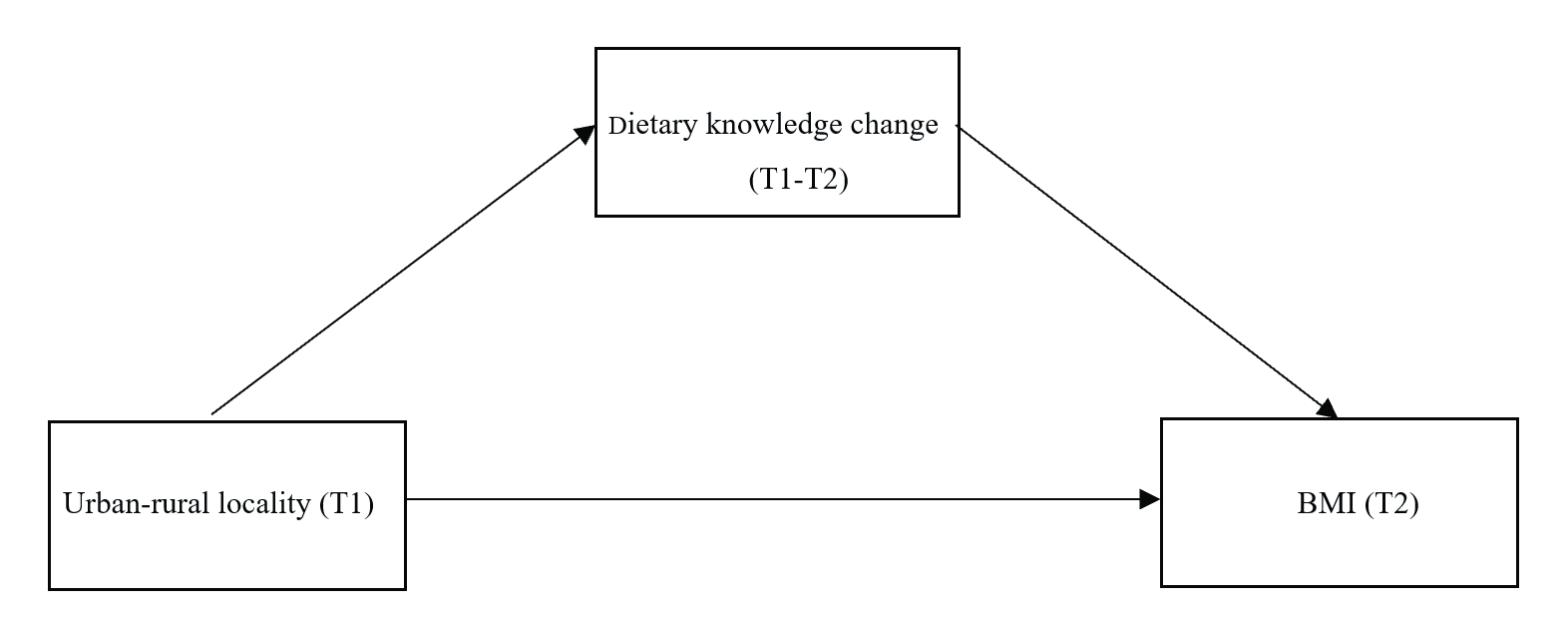
Mediation model 2: urban-rural locality (T1), dietary knowledge change (T1-T2), and BMI (T2).

**Figure 4:**
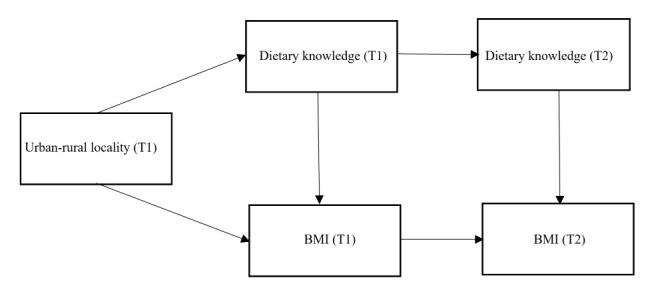
Mediation model 3: urban-rural locality (T1), dietary knowledge (T1 and T2), and BMI (T1 and T2).

We employed structural equation modelling (SEM) to test three different models that specified the relationship between urban-rural locality, dietary knowledge, and BMI. In model 1 (**Figure 2**), we focused on the cross-sectional associations between urban-rural locality, dietary knowledge, and BMI. We repeated SEM for T1 and T2, entering urban-rural locality as the independent variable, dietary knowledge as the mediator variable, and BMI as the dependent variable. Additionally, we entered the covariates at corresponding time points as control variables for both outcome and mediator variables. In model 2 (**Figure 3**), we restricted our focus to the longitudinal cohort and explored the associations between urban-rural locality (T1), dietary knowledge change (T1-T2), and BMI (T2). We entered urban-rural locality (T1) as the independent variable, dietary knowledge change (T1-T2) as the mediator variable, and BMI (T2) as the dependent variable. Meanwhile, we controlled the covariates (T1) for both outcome and mediator variables in these analyses. In Model 3 (**Figure 4**), we used the same longitudinal cohort to explore the pathways from urban-rural locality (T1) to BMI (T2). We established a two-phase model with multiple mediator variables (e.g. dietary knowledge at T1, BMI at T1, and dietary knowledge at T2), while the independent and dependent variable remained the same as in model 2. We further controlled the T1 covariates for dietary knowledge (T1) and BMI (T1) and the T2 covariates for dietary knowledge (T2) and BMI (T2).

For each model, we started by assessing the direct associations (e.g. urban-rural locality to BMI, urban-rural locality to dietary knowledge, and dietary knowledge to BMI) and calculated the indirect associations (e.g. urban-rural locality to dietary knowledge to BMI). Next, we stratified the participants by sex (men and women) and repeated these analyses. Finally, we conducted sensitivity analyses by omitting individual study covariates from the model to examine the effect of each on the results. We performed all SEM analyses with Stata 15 (Stata Corp), applying the robust maximum likelihood method. We evaluated SEM data fit for each model using comparative fit index (CFI), Tucker-Lewis index (TLI), and root mean square error of approximation (RMSEA), with values of CFI and TLI>0.90 and values of RMSEA<0.06 demonstrating adequate fit [30]. We considered α = 0.05 significant for all analyses.

## RESULTS

### Participant characteristics

The participants’ mean age increased from T1 to T2; there was also an increase in the proportion of married, female, non-smoking, and non-drinking participants, as well as the average score of dietary knowledge and the mean BMI. Urban and rural participants differed significantly by age, marital status, cigarette smoking, alcohol drinking, dietary knowledge, and BMI at T1 and T2. Compared to rural participants, urban participants were older, more likely to be married, non-smoking, and non-drinking, and they had better dietary knowledge and higher BMI (**Table 1**).

**Table 1:** General characteristics in the total study population and by urban-rural difference at different time points*

	T1				T2			
**Variables**	**Total (n = 8932)**	**Urban (n = 2794)**	**Rural (n = 6138)**	***P*-value†**	Total (n = 11 216)*	**Urban (n = 4515)**	**Rural (n = 6701)**	***P*-value†**
**Age in years**				0.001				0.001
Overall	47.71 (0.16)	50.03 (0.30)	46.73 (0.19)		53.30 (0.14)	54.26 (0.22)	52.66 (0.18)	
18-34	27.61 (0.11)	27.93 (0.21)	27.44 (0.13)		28.02 (0.12)	28.21 (0.20)	27.82 (0.14)	
35-49	42.14 (0.08)	42.24 (0.15)	42.14 (0.09)		42.90 (0.08)	42.76 (0.13)	43.17 (0.11)	
50-64	55.92 (0.08)	56.23 (0.17)	55.82 (0.09)		57.11 (0.07)	57.33 (0.10)	56.91 (0.09)	
65+	72.11 (0.15)	72.51 (0.23)	71.73 (0.20)		72.02 (0.11)	72.52 (0.18)	71.73 (0.14)	
**Sex, % women**	53.1	53.9	52.7	0.29	54.3	54.8	54.0	0.41
**Marital status, % married**	82.2	79.2	83.6	0.001	87.5	86.6	88.1	0.02
**Smoking, %**				0.001				0.001
Non-smoking	72.0	76.2	70.1		77.7	81.2	75.4	
1-3 cigarettes per day	1.6	1.6	1.6		1.4	1.6	1.3	
>3 cigarettes per day	26.4	22.2	28.3		20.8	17.2	23.3	
**Drinking, %**				0.001				0.001
Non-drinking	68.1	69.2	67.5		73.3	74.9	72.3	
Light drinking	16.9	17.8	16.5		15.6	16.0	15.3	
Heavy drinking	15.0	13.0	16.0		11.1	9.2	12.4	
**Dietary knowledge score**	39.00 (0.02)	39.55 (0.04)	38.74 (0.03)	0.001	43.43 (0.04)	44.05 (0.06)	43.01 (0.05)	0.001
**BMI in kg/m^2^**				0.008				0.001
Overall	23.07 (0.03)	23.55 (0.07)	22.86 (0.04)		24.28 (0.04)	24.50 (0.07)	24.13 (0.05)	
Undernutrition (<18.5)	17.48 (0.04)	17.29 (0.09)	17.55 (0.04)		17.35 (0.05)	17.31 (0.09)	17.38 (0.06)	
Normal (≥18.5 and <25)	21.87 (0.02)	22.11 (0.04)	21.77 (0.03)		22.26 (0.02)	22.40 (0.33)	22.17 (0.03)	
Overweight (≥25 and <30)	26.82 (0.03)	26.85 (0.05)	26.80 (0.04)		26.95 (0.02)	26.94 (0.03)	26.97 (0.03)	
Obese (≥30)	31.94 (0.11)	31.99 (0.18)	31.90 (0.14)		32.96 (0.27)	33.17 (0.56)	32.81 (0.25)	

BMI – body mass index

*Values are means (with standard errors) for continuous variables or percentages for categorical variables, for the total population and by urban-rural difference.

†*P*-values from χ^2^ test for categorical variables and one-way analysis of variance for continuous variables.

We also compared the descriptive statistics at T1 between participants included in the longitudinal cohort and those lost to follow-up (Table S2 in the **Online Supplementary Document**). We found significant differences in age, sex, marital status, dietary knowledge, and BMI; the included participants were younger and more likely to be female and married, and had lower dietary knowledge and higher BMI.

### Structural equation modelling

#### Model 1: Associations between urban-rural locality, dietary knowledge, and BMI at T1 and T2

We first performed the SEM at T1 for the overall sample and found a significant association between urban-rural locality and BMI (with rural individuals having lower BMI), between urban-rural locality and dietary knowledge (with rural individuals being more likely to report poorer dietary knowledge), and between dietary knowledge and BMI (with poorer dietary knowledge being associated with a lower BMI). Moreover, we observed a significant, indirect association between urban-rural locality to BMI through dietary knowledge. Next, we stratified the participants by sex (men vs women) and conducted SEM for each subgroup. We observed similar results to the overall analyses, except that urban-rural locality was no longer associated with BMI among women. We repeated these analyses at T2, both overall and stratified by sex and found similar results to T1, suggesting that these associations are robust over time (**Table 2**). Finally, we conducted a range of sensitivity analyses by omitting each individual study covariate one by one from the model, considering that some study covariates might interact with dietary knowledge and BMI. For both T1 and T2, exclusion of any single study covariate from the model did not yield results that were substantially different from the prior analyses. The model-data fit of Model 1 was adequate at T1 (CFI>0.99, TLI>0.99, RMSEA<0.01) and T2 (CFI>0.99, TLI>0.99, RMSEA<0.01).

**Table 2:** Direct and indirect associations between urban-rural locality, dietary knowledge, and BMI at different time points (T1 and T2)*

	T1			T2		
	**Men (n = 4188)**	**Women (n = 4744)**	**Overall (n = 8932)**	**Men (n = 5111)**	**Women (n = 6105)**	**Overall (n = 11 216)**
**Direct associations**						
Urban-rural locality to BMI	0.899 (0.681, 1.116)	0.149 (-0.073, 0.372)	0.481 (0.324, 0.638)	0.696 (0.455, 0.936)	0.161 (-0.065, 0.388)	0.421 (0.255, 0.588)
Urban-rural locality to dietary knowledge	0.618 (0.474, 0.762)	0.967 (0.831, 1.103)	0.804 (0.705, 0.903)	0.808 (0.563, 1.052)	0.857 (0.639, 1.075)	0.837 (0.674, 0.999)
Dietary knowledge and BMI	0.164 (0.119, 0.210)	0.130 (0.085, 0.176)	0.141 (0.108, 0.173)	0.055 (0.028, 0.082)	0.050 (0.024, 0.076)	0.051 (0.032, 0.070)
**Indirect associations**						
Urban-rural locality to dietary knowledge to BMI	0.101 (0.065, 0.138)	0.126 (0.079, 0.174)	0.113 (0.084, 0.1437)	0.044 (0.019, 0.070)	0.043 (0.018, 0.068)	0.043 (0.025, 0.060)

T1 – time point 1, T2 – time point 2, BMI – body mass index

*Values are regression coefficients (95% confidence interval), adjusted for age, sex (for overall analyses), education level, work status, marital status, cigarette smoking, and drinking of alcohol.

#### Model 2: associations between urban-rural locality (T1), dietary knowledge change (T1-T2), and BMI (T2)

In the overall sample for model 2, we found a significant association only between urban-rural locality (T1) and dietary knowledge change (T1-T2), with rural individuals exhibiting greater dietary knowledge change. Urban-rural locality (T1) was not associated with BMI (T2) and dietary knowledge change (T1-T2) did not predict BMI (T2). There was also a non-significant indication for dietary knowledge change (T1-T2) as a pathway from urban-rural locality (T1) to BMI (T2). We also stratified the participants by sex and examined these associations for each subgroup; we found similar results to the overall analyses, except for a significant association between urban-rural locality (T1) and BMI (T2) among men (**Table 3**). The results did not change in the sensitivity analyses after excluding the covariates one by one. The model-data fit of model 2 was adequate (CFI>0.99, TLI>0.99, RMSEA<0.01).

**Table 3:** Direct and indirect associations between urban-rural locality (T1), dietary knowledge change (T1-T2), and BMI (T2)*

	Men (n = 1810)	Women (n = 2263)	Overall (n = 4073)
**Direct associations**			
Urban-rural locality (T1) to BMI (T2)	0.718 (0.182, 1.253)	-0.364 (-0.795, 0.065)	0.100 (-0.235, 0.437)
Urban-rural locality (T1) to dietary knowledge change (T1-T2)	-0.779 (-1.292, -0.265)	-0.703 (-1.150, -0.255)	-0.704 (-1.041, -0.366)
Dietary knowledge change (T1-T2) to BMI (T2)	0.016 (-0.031, 0.064)	0.026 (-0.012, 0.066)	0.022 (-0.008, 0.053)
**Indirect associations**			
Urban-rural locality (T1) to dietary knowledge change (T1-T2) to BMI (T2)	-0.012 (-0.051, 0.025)	-0.018 (-0.049, 0.011)	-0.015 (-0.038, 0.006)

T1 – time point 1, T2 – time point 2, BMI – body mass index

*Values are regression coefficients (95% confidence interval), adjusted for age, sex (for overall analyses), education level, work status, marital status, cigarette smoking, and alcohol drinking.

#### Model 3: Associations between urban-rural locality (T1), dietary knowledge (T1 and T2), and BMI (T1 and T2)

Among all participants, we found a significant association between urban-rural locality (T1) and dietary knowledge (T1), between dietary knowledge (T1) and BMI (T1), between dietary knowledge at T1 andT2, and between BMI at T1 and T2, while we found a non-significant association between urban-rural locality (T1) and BMI (T1), and between dietary knowledge (T2) and BMI (T2). We did observe a significant indirect association from urban-rural locality (T1) to BMI (T2) through dietary knowledge (T1), BMI (T1), and dietary knowledge (T2). After stratifying by sex, we observed a significant association between urban-rural locality (T1) and BMI (T1) for men, while the indirect association from urban-rural locality (T1) to BMI (T2) was no longer significant for women; the other results did not change substantially (**Table 4**). We performed sensitivity analyses to assess the influence of each individual study covariate on the results and found no substantial changes from the above analyses. The model-data fit of model 3 was adequate (CFI = 0.96, TLI = 0.91, RMSEA = 0.04).

**Table 4:** Direct and indirect associations between urban-rural locality (T1), dietary knowledge (T1 and T2), and BMI (T1 and T2)*

	Men (n = 1810)	Women (n = 2263)	Overall (n = 4073)
Direct associations			
Urban-rural locality (T1) to BMI (T1)	0.914 (0.408, 1.421)	-0.242 (-0.601, 0.116)	0.242 (-0.017, 0.501)
Urban-rural locality (T1) to dietary knowledge (T1)	0.838 (0.606, 1.069)	1.045 (0.823, 1.266)	1.033 (0.873. 1.193)
Dietary knowledge (T1) to BMI (T1)	0.169 (0.101, 0.238)	0.141 (0.075, 0.206)	0.150 (0.102, 0.197)
Dietary knowledge (T1) to dietary knowledge (T2)	0.160 (0.073, 0.248)	0.134 (0.059, 0.209)	0.146 (0.089, 0.204)
BMI (T1) to BMI (T2)	0.817 (0.761, 0.874)	0.819 (0.782, 0.856)	0.819 (0.787, 0.850)
Dietary knowledge (T2) to BMI (T2)	0.014 (-0.029, 0.057)	0.000 (-0.032, 0.032)	0.006 (-0.020, 0.032)
**Indirect associations**			
Urban-rural locality (T1) to dietary knowledge (T1), BMI (T1), and dietary knowledge (T2) to BMI (T2)	0.866 (0.441, 1.291)	-0.077 (-0.368, 0.212)	0.323 (0.114, 0.533)

T1 – time point 1, T2 – time point 2, BMI – body mass index

*Values are regression coefficients (95% confidence interval), adjusted for age, sex (for overall analyses), education level, work status, marital status, cigarette smoking, and alcohol drinking.

## DISCUSSION

In line with previous research in developing countries [31-33], urban adults in our study tended to show consistently higher BMI than their rural counterparts in China (model 1). In contrast, the long-term effect of urban-rural locality on BMI was heterogenous and differed between men and women (model 2). Although persistent, the overall urban-rural disparity in BMI has decreased since 2004, especially in women. One possible interpretation for this is the divergent trends for the increase of BMI and obesity between men and women in urban and rural China. According to the findings from China Chronic Disease and Risk Factors Surveillance programme, the rise in BMI and obesity has slowed down in urban China since 2010, while continuously rising steadily in rural China, especially among women [3]. Similarly, many other LMICs have reported that BMI and obesity in rural populations (particularly women) are increasing faster than in urban areas [34]. These findings highlight that, although obesity remains severe in urban China, additional efforts are needed to prevent the rapid increase of BMI and obesity in rural adults, particularly women.

We also evaluated the association between dietary knowledge and BMI. In contrast with one earlier study that suggests no influence of dietary knowledge on BMI for Chinese adults [23], we found that dietary knowledge is significantly associated with BMI (Model 1). Such inconsistencies may come from differential strategies in calculating dietary knowledge. Instead of using the original 5-point Likert scale, Yu et al. [23] used a trichotomous grouping, thus losing statistical power to show a difference. Nevertheless, our findings were supported by an additional study conducted in another developing country [24]. Additionally, dietary knowledge change alone was insufficient to affect BMI in the long term (model 2) and the association between dietary knowledge and BMI appeared to lessen over time (model 3). While dealing with DBM in China, our findings provide support for dietary knowledge programmes to address undernutrition, as they are likely to increase BMI in the short term. However, more integrative and tailored programmes are called for to maintain a long-term effect.

Furthermore, we assessed the association between urban-rural locality and dietary knowledge. Consistent with other studies based on CHNS [19,35], we found rural individuals were associated with poorer dietary knowledge (model 1), but also that they tend to show greater change in dietary knowledge than those living in urban areas (model 2), which is an encouraging and novel finding. Besides alleviation of undernutrition, dietary knowledge is also beneficial to individuals’ dietary behaviours [36,37] and health [27,38]. Since urban-rural disparity in dietary knowledge remains, there is a need to further narrow this gap among Chinese adults.

Our study significantly contributes to current literature by examining dietary knowledge as a pathway from urban-rural locality to BMI. The indirect association between urban-rural locality and BMI through dietary knowledge was significant at each time point (model 1). Moreover, dietary knowledge appeared interactive with BMI over time, and this interaction was to be found a significant pathway from urban-rural locality to BMI in the long term, especially for men (model 3). Our analyses provide evidence in support of dietary knowledge as a mechanism in the association between urban-rural locality and BMI. Educational programmes focusing on improving dietary knowledge may be of use to China’s policymakers to narrow the gap of BMI differences between urban and rural locations, and consequently mitigate the burden of DBM (primarily undernutrition) in China.

Several limitations should be considered in interpreting our results. First, in addition to the specified covariates, a range of other factors might be associated with dietary knowledge and BMI [39]. However, we were unable to include these variables in our analyses due to data constraints. Furthermore, despite the relatively large sample size in each wave, we cannot exclude the possibility of sampling bias associated with attrition over a follow-up period that exceeded over 10 years (54% of the total participants at T1 were lost in the follow-up (Table S2 in the **Online Supplementary Document**)). Additionally, because the definition and categorisation of urban and rural locations varies by studies (especially between developing and developed countries), our findings should be interpreted cautiously. Finally, while we included the latest wave of CHNS data, it was conducted several years ago, so we lacked more recent data for analysing these associations.

## CONCLUSIONS

We used the SEM approach to assess the associations of urban-rural locality, dietary knowledge, and BMI in China. Urban-rural disparity in BMI persists among Chinese adults and is possibly mediated by dietary knowledge. Educational programmes for improving dietary knowledge could be a viable approach for China’s policymakers to improve the BMI of those suffering from undernutrition and mitigate the urban-rural disparity in BMI.

## Additional material


Online Supplementary Document
