# Supplementary material for: Urban-rural disparity in body mass index: is dietary knowledge a mechanism? Evidence from the China Health and Nutrition Survey 2004-2015
Source: J Glob Health. 2023 Jul 28;13:04064. doi: 10.7189/jogh.13.04064 (PMC10374270; doi:10.7189/jogh.13.04064)
Supplement: Online Supplementary Document [file jogh-13-04064-s001.pdf]

Table S1. Dietary knowledge questions and corresponding answers in the China Health and Nutrition Survey (CHNS)

No.	Do You Strongly Agree, Somewhat Agree, Somewhat Disagree or Strongly Disagree with this Statement, “Neutral” or “Unknown”?	True/False
Q1	Choosing a diet with a lot of fresh fruits and vegetables is good for one’s health	T
Q2	Eating a lot of sugar is good for one’s health	F
Q3	Eating a variety of foods is good for one’s health	T
Q4	Choosing a diet high in fat is good for one’s health	F
Q5	Choosing a diet with a lot of staple foods (rice and rice products and wheat and wheat products) is not good for one’s health	T
Q6	Consuming a lot of animal products daily (fish, poultry, eggs and lean meat) is good for one’s health	F
Q7	Reducing the amount of fatty meat and animal fat in the diet is good for one’s health	T
Q8	Consuming milk and dairy products is good for one’s health	T
Q9	Consuming beans and bean products is good for one’s health	T
Q10	Physical activities are good for one’s health	T
Q11	Sweaty sports or other intense physical activities are not good for one’s health	F
Q12	The heavier one’s body is, the healthier he or she is	F

Table S2. General characteristics at T1 between participants included in the longitudinal analyses and those excluded

Variables	Total* (n = 8,932)	Longitudinal		
		Included* (n = 4,073)	Excluded* (n = 4,859)	*P*-value†
**Age (years)**				***P* = 0.006**
Overall	47.71 (0.16)	47.25 (0.19)	48.14 (0.25)	
18-34	27.61 (0.11)	29.08 (0.16)	26.76 (0.14)	
35-49	42.14 (0.08)	42.32 (0.11)	41.87 (0.12)	
50-64	55.92 (0.08)	55.78 (0.11)	56.11 (0.13)	
65+	72.11 (0.15)	69.16 (0.18)	73.05 (0.18)	
**Sex (% women)**	53.1	55.6	51.1	***P* < 0.001**
**Marital status (% married)**	82.2	89.7	75.9	***P* < 0.001**
**Smoking (%)**				*P* = 0.52
Non-smoking	72.0	71.9	72.1	
1-3 cigarettes per day	1.6	1.4	1.7	
> 3 cigarettes per day	26.4	26.7	26.2	
**Drinking (%)**				*P* = 0.13
Non-drinking	68.1	67.3	68.7	
Light drinking	16.9	16.8	17.0	
Heavy drinking	15.0	15.9	14.3	

**Dietary knowledge（score）**	39.00 (0.02)	38.93 (0.03)	39.05 (0.03)	***P* = 0.01**
**BMI (kg/m²)**				***P* = 0.02**
Overall	23.07 (0.03)	23.17 (0.05)	23.00 (0.05)	
Undernutrition (<18.5）	17.48 (0.04)	17.58 (0.05)	17.43 (0.05)	
Normal (≥18.5 and <25)	21.87 (0.02)	21.89 (0.03)	21.85 (0.03)	
Overweight (≥25 and <30)	26.82 (0.03)	26.83 (0.04)	26.81 (0.04)	
Obese (≥30)	31.94 (0.11)	31.98 (0.18)	31.91 (0.14)	

*Values are means (with standard errors) for continuous variables or percentages for categorical variables, for the total population and by urban-rural difference.

†P-values are chi-squared test for categorical variables, and one-way analysis of variance continuous variables.
